# Transforming Cancer Therapy: Unlocking the Potential of Targeting Vascular and Stromal Cells in the Tumor Microenvironment

**DOI:** 10.1158/0008-5472.CAN-24-4744

**Published:** 2025-04-02

**Authors:** Lu Liu, Yuheng Zhang, Hanyu Liu, Jian Yang, Qi Tian, Nareekarn Chueakula, Saravana Ramasamy, Navin Kumar Verma, Christine Cheung, Anjali P. Kusumbe

**Affiliations:** 1 Tissue and Tumor Microenvironments Lab, Cancer Discovery and Regenerative Medicine Program, Lee Kong Chian School of Medicine, Nanyang Technological University, Singapore, Singapore.; 2 Lee Kong Chian School of Medicine, Nanyang Technological University, Singapore, Singapore.; 3 Multidisciplinary Institute of Ageing (MIA-Portugal), Coimbra, Portugal.

## Abstract

The tumor microenvironment (TME) orchestrates cancer progression by fostering a complex interplay between cancer cells and the surrounding cellular and acellular elements. Through dynamic interactions with cancer cells, vascular and stromal cells not only promote tumor growth but also enhance metastatic potential and restrict therapeutic responses. Vascular and stromal cells play a critical role in regulating epithelial–mesenchymal transition (EMT) and sustaining resistance pathways, making them compelling targets for innovative therapies. This review delves into the vascular and stromal components of the TME, their contributions to EMT and resistance mechanisms, and emerging strategies to target these interactions for improved cancer therapy outcomes.

## Introduction

Over recent years, advancements in cancer therapy have led to extended survival rates; however, therapeutic success remains variable and highly influenced by tumor types, genetic profile, and individual patient factors (1). Metastasis—the process by which malignant cells spread from the primary tumor to distant sites—is a defining characteristic of cancer and the leading cause of cancer-related mortality (2). Metastatic progression requires the travel of cancer cells and their adaptation and constant evolution through continuous and complex interactions with their surrounding niches, eventually allowing them to change their normal behavior and become aggressive (3). Despite the critical role of metastasis in cancer’s lethality, its underlying mechanisms are still not fully understood.

The epithelial–mesenchymal transition (EMT) is a biological process in which epithelial cells lose their original characteristics and acquire mesenchymal properties (4). This transition enhances the cells' ability to move and invade, thereby contributing to metastatic spread (4). As tumor cells undergo EMT, they gain the ability to invade nearby tissues and enter circulation, enabling dissemination to distant organs (5). Intriguingly, once they reach a new site, these cells may undergo a reverse process, known as the mesenchymal–epithelial transition, which facilitates their colonization and growth in secondary locations (5). The dual ability of cells to shift between epithelial and mesenchymal states underscores the influence of the tumor microenvironment (TME), which provides external signals that drive these transitions (6, 7).

**Figure 1.**
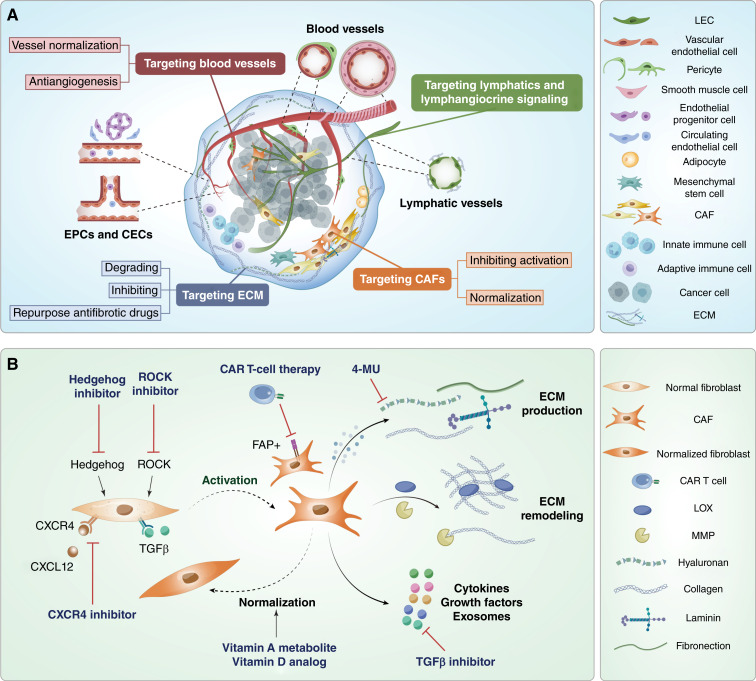
Vascular and stromal components with targeting strategies in the TME. **A,** The TME is composed of vascular cells such as lymphatic and blood endothelial cells and stromal cells like CAFs, pericytes, and adipocytes, along with their ECM components. In addition to the vascular and stromal cells, other cells such as immune cells also form the TME. Vascular and stromal cells with the TME drive tumor growth, metastasis, and therapeutic resistance. Emerging strategies target blood vessels, ECM, CAFs, and lymphatics to improve cancer therapy outcomes. **B,** CAFs can be targeted using various strategies, such as aiming FAP^+^ CAF, interfering with CAF activation using TGFβ, CXCR4, ROCK signaling, and Hedgehog signaling inhibitors, influencing CAF signaling using TGFβ, CAF normalization using vitamin A metabolites and vitamin D analogs, which are either FDA approved or currently being evaluated in clinical trials, or inhibiting ECM production by the compound 4-MU, a clinically approved hyaluronan synthesis inhibitor. CEC, circulating endothelial cell; CXCL, chemokine (C-X-C motif) ligand; CXCR4, C-X-C chemokine receptor type 4; EPC, endothelial progenitor cell; 4-MU, 4-methylumbelliferone.

The TME is a multifaceted ecosystem encompassing diverse cell types, including vascular, stromal, and immune cells (5). The vascular compartment comprises various cell types, such as endothelial cells of blood vessels, lymphatic endothelial cells (LEC), and pericytes. Additionally, the vascular microenvironment may include other perivascular cells, such as mesenchymal stem cells, certain stromal cells, and perivascular adipocytes. In the stromal compartment, cancer-associated fibroblasts (CAF) are the key players (Fig. 1A). Beyond these cellular components, the extracellular matrix (ECM) also forms an integral part of the TME. Details of different cellular components of the TME are reviewed here (8).

Given the crucial role of vascular and stromal cell types in driving EMT, metastasis, and resistance to standard therapies, targeting its components presents a promising therapeutic approach (Fig. 1A; ref. 5). This review discusses the key players in the TME that are vascular and stromal cells and highlights the current therapies aimed at vascular and stromal cell targeting. Additionally, it discusses the challenges and future directions for targeting the vascular and stromal cells in cancer therapies.

## Role of Vascular and Stromal Cells in EMT

Vascular and stromal cells release cytokines and chemokines that signal to nearby tumor cells through paracrine communication. These interactions can lead to EMT, a process that enhances the invasive and metastatic properties of the cancer cells. EMT involves a cascade of cellular changes governed by EMT-inducing transcription factors (EMT-TF), including TWIST-related protein 1 and 2, ZEB1, ZEB2, SNAIL, and SLUG (9, 10). These transcriptional regulators are influenced by various TME-derived signaling pathways that alter gene expression and promote cellular transformation (11). One prominent pathway is the TGFβ pathway, which activates EMT-TFs through both SMAD-dependent and -independent routes (12). TGFβ functions as a tumor suppressor in early stages but as a promoter in advanced stages of cancer, further complicating its targeting (13, 14). In normal and premalignant cells, TGFβ primarily functions as a tumor suppressor by inhibiting proliferation, inducing apoptosis, and preserving genome stability (15). However, tumor cells can evade or adapt to its suppressive effects, leveraging promotional roles of TGFβ to gain a growth advantage and drive processes like EMT, facilitating their migration, invasion, intravasation, and extravasation (16). Additionally, TGFβ fosters a tumor-supportive TME by activating CAFs, promoting angiogenesis, producing ECM, and suppressing antitumor immunity (14).

Other important pathways influencing EMT include the Wnt, Notch, EGF, insulin-like growth factor 1, and hepatocyte growth factor (HGF; ref. 17). Another influential factor in EMT induction is hypoxia, a condition commonly found in solid tumors because of limited blood supply (18). Hypoxia-induced EMT is largely regulated by hypoxia-inducible factor-1α (HIF1α), a master regulator activated under low oxygen levels (19). HIF1α promotes EMT by suppressing E-cadherin and activating EMT-TFs, such as Twist and ZEB1, enhancing cell motility and invasion (20, 21).

HIF1α also interacts with other EMT-related pathways to reinforce the EMT process (19). For example, it upregulates TGFβ1/2, which in turn enhances HIF1α stability through feedback mechanisms that further drive EMT (22, 23). Additionally, HIF1α collaborates with the Notch signaling coactivator MAML1 to increase expression of *Slug* and *Snail*, thereby promoting cancer cell migration and invasion (24). HIF1α also regulates noncoding RNAs, such as long noncoding RNA UCA1, which is released from hypoxic cells via exosomes and facilitates EMT in recipient tumor cells (25). The interactions between hypoxia, TME signaling, and EMT underscore the adaptability of cancer cells within a hostile microenvironment (26). By activating a network of signaling pathways, the vascular and stromal cells foster EMT, enhancing cancer cell plasticity, invasiveness, and metastatic capability, and ultimately posing challenges for effective cancer therapy.

## Role of CAFs in EMT

CAFs, the most prevalent stromal cells within the TME, play a central role in promoting tumor progression by supporting cellular growth, angiogenesis, ECM remodeling, and immune suppression (Supplementary Table S1; ref. 27). CAFs are heterogeneous, functionally diverse, and metabolically active population of cells and drive various protumorigenic processes (28). Through their secreted factors and signaling capabilities, CAFs have been shown to initiate the EMT, enhance the invasive and metastatic potential of cancer cells, and promote resistance to antitumor vaccination and immunotherapy, while suppressing antitumor immunity (27, 29).

In normal tissues, fibroblasts produce ECM components and enzymes that maintain tissue structure and aid in wound healing (30). However, CAFs actively remodel the ECM in a way that promotes tumor growth (31). CAFs are responsible for synthesizing ECM proteins like collagen, fibronectin, and proteoglycans, contributing to matrix stiffening—a process that promotes EMT through mechanotransduction pathways, including Hippo and ROCK signaling (Fig. 1B; refs. 31–33). In addition, CAFs produce enzymes like lysyl oxidase (LOX), which cross-links collagen to stiffen the matrix, and matrix metalloproteinases (MMP), which degrade ECM components to facilitate cancer cell invasion (Fig. 1B; refs. 34, 35). By adjusting the ECM’s structure, CAFs create a mechanically supportive environment for cancer progression.

CAFs also release cytokines, exosomes, and growth factors, further driving cancer progression and other TME components, including immune cells (Fig. 1B; ref. 36). For example, cytokines like TGFβ, IL6, and CXCL9 modulate T-cell responses, whereas CAF-derived VEGF supports angiogenesis (37).

A key cytokine secreted by CAFs is TGFβ, a potent inducer of EMT (29). Studies have demonstrated that breast cancer cells exposed to CAF-conditioned media show increased motility, reduced *E-cadherin* expression, and elevated levels of EMT markers such as vimentin, fibronectin, and MMPs (MMP2 and MMP9; refs. 27, 38). Notably, this EMT effect can be reversed by TGFβ-neutralizing antibodies or inhibitors, emphasizing the critical role of TGFβ in CAF-mediated EMT (39). In urinary bladder cancer, TGFβ from CAFs induces EMT by regulating ZEB2 through both transcriptional activation and upregulation of ZEB2NAT, a long noncoding RNA that promotes ZEB2 translation (40). IL6, another cytokine secreted by CAFs, is also integral to EMT induction, particularly through the JAK2/STAT3 pathway, enhancing the EMT process in lung, liver, and bladder cancers (41–43). IL6 not only triggers EMT directly but also amplifies TGFβ signaling, creating a feedback loop that strengthens EMT and contributes to invasiveness, metastatic spread, and chemoresistance (44, 45).

In addition to TGFβ and IL6, CAFs release a variety of growth factors that facilitate EMT. For example, CAF-conditioned media often contain elevated levels of EGF, HGF, and FGF2, which have been shown to induce EMT in endometrial cancer cells and promote metastasis *in vivo* (46). Furthermore, HGF from CAFs activates IL6 receptors in gastric cancer cells, whereas CAF-secreted IL6 increases the expression of the HGF receptor c-Met, illustrating a complex feedback loop between CAFs and cancer cells (47). CAFs also secrete FGF1, which activates the MEK/ERK pathway to upregulate EMT markers like Snail and MMP3 in ovarian cancer cells, underscoring the multifaceted role of CAFs in driving EMT (48). This network of CAF-derived factors reveals the influence of CAFs on EMT through multiple signaling pathways, highlighting the critical role of CAFs as enablers of cancer progression and suggesting potential targets for therapeutic intervention.

### CAF subtypes

The advancement of technologies like single-cell RNA sequencing and genetic mouse models has significantly advanced the understanding of CAF heterogeneity (Supplementary Table S2). Although there is no universally accepted classification system, CAFs are widely categorized into three primary subtypes across different cancers (49, 50). Myofibroblastic CAFs (myCAF) are characterized by abundant ECM transcript expression and are central to ECM secretion and remodeling. Inflammatory CAFs (iCAF), marked by upregulated cytokines and growth factors, contribute to the inflammatory TME, whereas antigen-presenting CAFs, distinguished by high expression of MHC class II molecules, influence immune responses. Additional CAF subtypes have also been identified, including vascular CAFs, enriched in vascular development–related transcripts; epithelial-to-mesenchymal–like CAFs expressing EMT markers; lipofibroblast CAFs involved in lipid metabolism; progenitor CAFs, which are immature cells capable of differentiating into other CAF types; and matrix CAFs, which are significant collagen secretors (51, 52). iCAFs/myCAFs and mesothelial CAFs are also reported (51). CAF subtype dynamics vary during cancer progression, with progenitor CAFs and iCAFs being prevalent in early stages, whereas myCAFs and matrix CAFs dominating later stages (52). Spatially, subtypes are differentially localized; for example, in pancreatic cancer, myCAFs are proximal to cancer cells, whereas iCAFs are found more distantly (53), reflecting their temporal and spatial plasticity. Intriguingly, certain CAF populations identified in murine models of bladder, colon, and pancreatic cancers can suppress tumor growth, and some CAF subtypes exhibit both tumor-promoting and tumor-suppressing roles (54). These findings highlight the potential therapeutic strategy of inducing a “stromal switch,” converting protumor CAFs into tumor-suppressing CAFs, as a promising avenue for anticancer therapy (55).

### CAFs and aging

Most cancers occur in individuals over 60 years of age or the frequency increases with aging. Aging tissues are characterized by vascular attrition (56, 57), accumulation of senescent cells, and increase in fibroblasts (58). In aging tissues, pericytes have been shown to differentiate into fibroblasts (59). Age-related changes in stromal cells are associated with tumor development and progression (60). Senescent fibroblasts exhibit a senescence-associated secretory phenotype and proinflammatory molecules. The senescence-associated secretory phenotype enhances senescence through autocrine signaling and induces senescence of neighboring cells via paracrine signaling, leading to increased inflammation (61, 62). Moreover, during aging, there is a decline in immune surveillance and reduction in the ability to clear senescent cells. This chronic inflammation can cause cellular and DNA damage, leading to fibroblast senescence (63). Cancer therapies, including radiotherapy, chemotherapy, and anticancer drugs, can also accelerate the accumulation of senescent fibroblasts (61). Although senescent stromal fibroblasts are clearly associated with tumors, the underlying mechanisms remain elusive. Further research is needed on specific markers to distinguish between benign and tumor-associated senescent fibroblasts and between age-associated fibroblasts versus CAFs.

### Targeting CAFs

Given their central role, targeting CAFs has become a key strategy in anticancer therapy development (Fig. 1B; ref. 64). Many therapies focus on fibroblast activation protein (FAP), which is overexpressed in more than 90% of cancers (65). FAP-positive CAFs are linked to immune suppression in both animal models and human samples (66, 67). Agents targeting FAP, such as RO6874281, sibrotuzumab, and FAP-IL2v, are undergoing early-phase clinical trials for advanced solid tumors. Additionally, an oral DNA vaccine targeting FAP has shown promise in enhancing CD8^+^ T cell–mediated CAF killing, reducing tumor growth and metastasis in preclinical models (68). FAP-specific chimeric antigen receptor (CAR) T cells have also demonstrated effectiveness in eliminating FAP^+^ CAFs and curbing tumor growth in mouse models of lung, mesothelioma, and pancreatic cancers (69–71).

Despite initial promise, some FAP-targeting approaches, such as the monoclonal antibody sibrotuzumab, have shown limited efficacy because of FAP’s expression in normal tissues (72, 73), in which it plays roles in tissue homeostasis. This highlights the need for greater specificity in targeting CAFs (74). Alternative strategies include inhibiting pathways like FGFR, hedgehog, ROCK, NF-κB, TGFβ, and CXCR4, which are pivotal in CAF activation (Fig. 1B; ref. 31). FGFR inhibitors, such as erdafitinib, have shown significant success in FGFR-altered urothelial carcinoma and have received FDA approval (75). Hedgehog pathway inhibitors like vismodegib and sonidegib have also achieved FDA approval for basal cell carcinoma but have shown mixed results in combination therapies for other cancers (31, 76).

Stellate cells are known to regulate blood flow and vitamin A metabolism and contribute to liver and pancreatic fibrosis. Quiescent stellate cells produce low levels of ECM. During tumorigenesis, stellate cells transdifferentiate into myofibroblast-like cells, contributing to CAFs in the TME; those activated stellate cells are highly proliferative and secrete high levels of ECM (Fig. 1B; ref. 77). Vitamin D analogs, such as paricalcitol, have been shown to revert CAFs to their quiescent stellate cell state in pancreatic cancer models and are being tested in early-phase clinical trials for advanced solid tumors (31). An active metabolite of retinol, all-trans retinoic acid, can convert CAFs into a less active, quiescent state, showing lower expression of αSMA, IL6, and ECM. It inhibits the migration and EMT of pancreatic tumor cells (78–81). In clinical trials, all-trans retinoic acid has been used in combination with other drugs and has shown promising prospects in treating recurrent/metastatic adenoid cystic carcinoma of the head and neck, non–high-risk acute promyelocytic leukemia, and recurrent isocitrate dehydrogenase–mutant glioma (82, 83). Other targeted strategies involve blocking TGFβ, CXCR4, or ROCK signaling, with inhibitors like galunisertib, AMD3100, and AT13148 showing promising results in preclinical and early-phase clinical trials (84–86). For example, TGFβ inhibitors combined with chemoradiotherapy have improved response rates in solid tumor patients, whereas CXCR4 inhibitors have enhanced stem cell mobilization in hematologic malignancies (84, 85).

Despite recent advancements, significant challenges remain in targeting CAFs. CAF subtypes exhibit considerable heterogeneity and genetic instability, complicating therapeutic strategies (31). There have been some reports that CAFs may show genetic instability and chromosomal and/or gene copy-number alterations in breast cancer, prostate cancer, colorectal cancer, and ovarian cancer (87–90). For instance, CD10^+^ and GPR77^+^ CAFs confer chemotherapy resistance in breast cancer models, whereas aged fibroblasts with altered lipid metabolism drive therapy resistance in melanoma (91, 92). A deeper understanding of CAF biology, including subtype-specific roles and changes during cancer progression, is crucial to developing effective, tailored therapies. Future research should focus not only on CAF depletion but also on reprogramming CAFs to a quiescent state and targeting CAF-derived factors to improve therapeutic outcomes.

## Targeting of Vascular and Stromal ECM for Cancer Therapies

The ECM derived from vascular and stromal cells plays a critical role in regulating the TME and cancer progression (93, 94). Increased ECM stiffness in the surrounding tissue promotes EMT in cancer cells, driving invasiveness, stemness, and metastasis (33, 94). Additionally, the expression of ECM-related genes, such as *SPARCL1* and *TWIST*, is associated with poor prognosis and heightened resistance to therapy in various cancers (95, 96). Excessive ECM accumulation activates integrin and focal adhesion kinase (FAK) signaling, leading to reduced apoptosis, enhanced pro-survival pathways, and increased chemoresistance. Furthermore, abnormal ECM buildup can physically obstruct drug delivery, limiting therapy efficacy (97). Given these critical roles of ECM in cancer, targeting its components has become a key focus in TME research. Three main strategies are being explored to target ECM: (i) degrading ECM components, (ii) directly inhibiting the synthesis of new ECM components, and (iii) repurposing antifibrotic drugs. These approaches aim to disrupt the tumor's protective matrix, thereby improving therapy outcomes.

### Degrading ECM

Degrading different components of the ECM is a strategy to improve the distribution of drugs. Enzymes like hyaluronidases and collagenases are used for ECM breakdown (98). One such approach involves PEGylated human hyaluronidase (PEGPH20), which is often combined with other therapies in clinical trials for advanced solid tumors. In a phase II trial (NCT01839487) of patients with pancreatic cancer, the combination of PEGPH20 with nab-paclitaxel and gemcitabine showed a significant improvement in progression-free survival (99). However, this therapy was associated with frequent grade 3/4 adverse events, such as muscle spasms, neutropenia, and myalgia (99).

Despite these promising findings, subsequent clinical trials have yielded mixed results. A phase III study (NCT02715804) involving the same combination failed to show benefit in patients with stage IV pancreatic cancer and with high hyaluronan levels (100). Additionally, a phase Ib/II trial (NCT01959139) combining PEGPH20 with FOLFIRINOX chemotherapy showed increased toxicity and unfavorable effects in patients with metastatic pancreatic adenocarcinoma (101).

### Inhibiting ECM

Inhibiting the *de novo* synthesis of ECM components involves targeting key signaling pathways, such as HIF1α or TGFβ, that drive ECM production, or by blocking enzymes responsible for modifying and secreting these components. One such enzyme is LOX, which is critical for the stabilization of collagen networks. However, efforts to inhibit LOX activity have had mixed results. For example, the combination of the LOXL2 antibody simtuzumab with FOLFIRI chemotherapy or gemcitabine did not show significant clinical benefit in patients with pancreatic cancer (NCT01472198) or colorectal carcinoma (NCT01479465; refs. 102, 103). These findings highlight the challenge of targeting ECM synthesis in cancer therapy. In addition to these two studies, many others have explored strategies to target the LOX family, either directly with inhibitors or indirectly by modulating its expression or activity. These approaches have been comprehensively reviewed (104).

Fibronectin, a large glycoprotein in the ECM, interacts with other matrix molecules and cell receptors such as integrins to drive tumor growth, angiogenesis, invasion, and metastasis (105–107). Studies have shown that when mammary epithelial cells are exposed to fibronectin, they undergo EMT, upregulating EMT markers via the ERK/MAPK signaling pathway (108). Increased fibronectin levels have also been linked to EMT activation in renal cell carcinoma and soft-tissue sarcoma, in which it upregulates the EMT-TF Slug and promotes metastasis (109). In breast cancer, fibronectin binding to integrins activates FAK, triggering EMT and upregulating N-cadherin and vimentin, leading to enhanced cell migration and invasion (110). Interestingly, FAK inhibitors, such as PF-00562271, have shown efficacy in preclinical models and are currently under clinical investigation, indicating the potential of targeting fibronectin–FAK interactions as a therapeutic approach in cancer (Supplementary Table S3; refs. 111–113).

Hyaluronan, a crucial ECM component, plays a significant role in driving EMT (94). Elevated levels of hyaluronan synthase 3 in lung cancer cells have been shown to induce an EMT-like state, characterized by increased invasiveness and enhanced activity of MMP2 and MMP9 (114). Additionally, the interaction between hyaluronan and the cell surface receptor CD44 initiates LOX production, which subsequently elevates Twist expression, triggering EMT (115). TGFβ has also been found to induce EMT via upregulation of HAS2, whereas suppressing HAS2 reduces TGFβ-driven EMT by approximately 50%, underscoring hyaluronan’s role in this process (116). Interestingly, the compound 4-methylumbelliferone, a clinically approved hyaluronan synthesis inhibitor, has demonstrated the ability to reduce cancer cell growth, migration, and metastatic spread in both pancreatic and lung cancers (Fig. 1B; Supplementary Table S3; refs. 117, 118). Although more clinical validation is needed, these findings point to the potential of 4-methylumbelliferone as a promising therapeutic approach.

ECM remodeling enzymes regulate essential processes in cancer cells. MMPs, plasminogen activators, and cathepsins control ECM protein hydrolysis, which drives cancer cell invasiveness and promotes distant metastases (119–121). Furthermore, glycolytic enzymes such as heparanase and hyaluronidase play unique roles in tumor progression by cleaving heparan sulfate and hyaluronan, respectively (122, 123). Given the established roles of these matrix enzymes, they are rational pharmacologic targets for cancer therapy (124); however, most of the clinical efforts failed because of lack of specificity and other related impact as discussed in Supplementary Text.

### Repurposing of drugs with antifibrotic properties

Repurposing existing FDA-approved drugs for new indications offers a promising strategy in cancer therapy (125). This approach bypasses many challenges associated with traditional drug development, such as dose optimization and safety profiling, making it a more cost-effective, time-efficient, and lower-risk option for clinical trials (126). Antifibrotic agents such as metformin, losartan, and pirfenidone are being explored for advanced cancers. For example, in a phase II clinical trial (NCT01821729), the combination of losartan, FOLFIRINOX, and chemoradiotherapy showed improvements in patients with pancreatic cancer (127). However, in a phase III trial (NCT01101438), no improvement was seen in disease-free survival in high-risk patients with breast cancer when metformin was used along with standard treatment (128). Although other repurposed drugs hold potential for co-treatment in pancreatic cancer, further studies are needed to better understand the safety and efficacy of these agents in clinical settings (126).

## Tumor-Associated Blood Vessels

The developed tumor vasculature supports blood supply to cancer cells. However, the new rapidly formed blood vessels are immature and excessively branched, leading to their abnormal organization and heterogenicity, exhibiting notable structural and functional abnormalities, including increased vascular permeability (129). These issues arise because of factors such as disrupted endothelial junctions, elevated endothelial cell proliferation, excessive ECM deposition, and compromised pericyte coverage (130). These vascular defects contribute to insufficient oxygen delivery, creating a hypoxic microenvironment that is closely linked to enhanced tumor aggressiveness (130, 131). The hypoxic TME inhibits the differentiation and maturation of tumor-associated dendritic cells, causes apoptosis of tumor-resident and -infiltrating T cells, and inhibits T-cell effector function (132). Additionally, dysfunctional blood vessels hinder proper vascular perfusion, which may suppress the infiltration of cytotoxic T lymphocytes and other immune effector cells, limiting the body's ability to fight cancer (133, 134). Antiangiogenic therapies have been developed to target these abnormal tumor blood vessels (135). These therapies focus on selectively regressing immature vessels while promoting vascular normalization—a process that improves vessel integrity and function. This approach aims to not only reduce tumor growth by limiting nutrient and oxygen supply but also enhance the effectiveness of immune therapies and improve overall tumor therapy outcomes (134, 136).

Tumor-associated blood vessels are closely linked to distant metastasis by contributing to the formation of premetastatic niches through increased vascular permeability, angiogenesis, and pro-angiogenic molecules. Distant metastasis is the leading cause of death in patients with cancer, involving the spread of cancer cells from the primary site to distant organs. Primary tumors induce early changes in the distant microenvironments termed as premetastatic niche of secondary organs before metastases (137). Premetastatic niches include increased vascular permeability, angiogenesis, and inflammation (138–140). Tumor-derived exosomes also contribute to the development of premetastatic niches through proangiogenic molecules, such as VEGF, MMPs, and miRNAs (141). CAFs and LECs in the premetastatic niches promote tumor cell recruitment and invasion and promote angiogenesis (142, 143). Antiangiogenic therapy has shown potential in combating tumor metastasis. Anti-VEGF drugs including bevacizumab, sunitinib, and VEGF receptor inhibitors such as sorafenib and ramucirumab inhibit the activation of VEGF signaling and block angiogenesis (144).

Endothelial progenitor cells contribute to tumor vascularization and progression by migrating to sites of neovascularization, differentiating into endothelial cells and shaping the TME, whereas circulating endothelial cells serve as biomarkers for tumor vascularization. They are potential therapeutic targets and delivery vehicles in cancer treatment (Supplementary Text; refs. 145, 146).

## Targeting Tumor Vasculature

**Figure 2.**
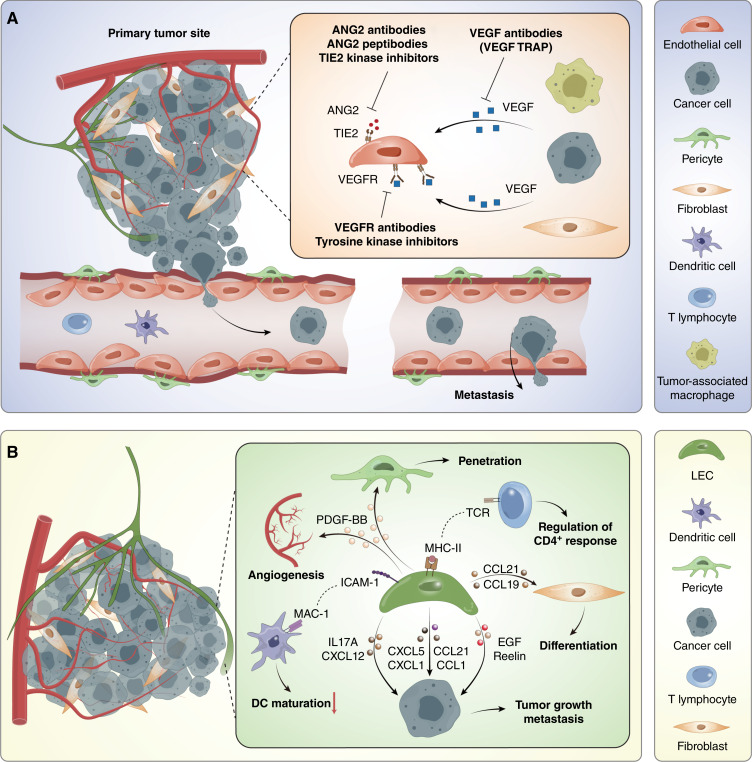
Therapeutic targeting of tumor vasculature. **A,** Inhibition of VEGF and/or VEGFR is the most used antiangiogenic strategy accomplished with several FDA-approved agents, such as anti-VEGF and VEGF-TRAP (VEGF decoy receptors), and/or VEGFR-specific antibodies and tyrosine kinase inhibitors, respectively. Alternatively, ANG2–TIE2 inhibitors currently being tested in the clinic can also be used to promote antiangiogenesis. Drugs targeting tumor vasculature, either FDA approved or those being evaluated at different stages of clinical development drugs, are referenced in the text. **B,** There are complex interactions between lymphatic vessels with the surrounding stromal cells, immune cells, and cancer cells. LECs secrete PDGF-BB to drive angiogenesis. LECs also exert immunosuppressive effects in the TME. They regulate CD4^+^ T-cell responses via MHC-II–dependent pathways while suppressing dendritic cell (DC) maturation and T-cell activation through ICAM-LEC–derived CCL19 and CCL21 and enhance fibroblast differentiation, whereas signaling molecules like ILs, CXCLs, CCLs, EGF, and Reelin support tumor growth and metastasis. ANG2, angiopoietin-2; CCL, chemokine (C-C motif) ligand; CXCL, chemokine (C-X-C motif) ligand; ICAM-1, intercellular adhesion molecule 1; MAC-1, macrophage-1 antigen; TCR, T-cell receptor; TIE2, tyrosine kinase with immunoglobulin and EGF–like domains 2.

Targeting tumor vasculature remains a cornerstone of cancer therapy, with many clinical studies exploring the combination of antiangiogenic therapies with chemotherapy or immunotherapy (Fig. 2A; Supplementary Table S3; refs. 147, 148). VEGFA, a key regulator of tumor angiogenesis, activates VEGFR-2 to upregulate VEGFR-3 and DLL4 in tip cells, while triggering Notch signaling (149). It also activates p38/MAPK and PI3K/AKT, promoting proliferation and migration of endothelial cells (149). Additionally, VEGFA recruits bone marrow–derived circulating endothelial progenitor cells (150). VEGFA antibodies reduce tumor vasculature, which led to the popular hypothesis that anti-VEGFA therapy could starve tumors by inhibiting angiogenesis (150). However, subsequent research revealed a more complex effect, showing that anti-VEGFA therapy can also transiently normalize tumor vasculature, improving perfusion through reduced vascular density, increased blood flow, decreased permeability, and enhanced pericyte recruitment (151). This supports combining anti-VEGFA therapy with other anticancer drugs to enhance drug delivery. VEGFA also suppresses immunity by modulating dendritic cells, myeloid-derived suppressor cells, and tumor-associated macrophages, promoting regulatory T-cell accumulation and T-cell suppression, facilitating immune escape (152). Anti–VEGF-A/VEGFR agents like bevacizumab and sunitinib can counteract this immune suppression and restore antitumor immunity (152).

Bevacizumab, an anti-VEGFA monoclonal antibody, has demonstrated notable anticancer effects in several advanced solid tumors. It has gained FDA approval for use both as monotherapy and in combination with other therapies (130, 153). Currently, more than 1,000 clinical trials are investigating bevacizumab, often in conjunction with immunotherapeutic agents, underscoring its importance in improving clinical outcomes by targeting tumor vasculature (37). For instance, a meta-analysis revealed that bevacizumab combined with chemotherapy enhanced overall response rate, progression-free survival, and overall survival in patients with metastatic colorectal cancer compared with chemotherapy alone (154). However, results are mixed; for example, a phase III trial (NCT00528567) found no improvement in overall survival when bevacizumab was combined with anthracycline- or taxane-based chemotherapy in patients with triple-negative breast cancer (155).

Other antiangiogenic agents include aflibercept, a decoy receptor that binds VEGFA, VEGFB, and placental growth factor. Aflibercept in combination with FOLFIRI chemotherapy has demonstrated significant benefits in metastatic colorectal cancer, leading to FDA approval (156). Similarly, ramucirumab, a monoclonal antibody targeting VEGFR-2, has shown efficacy as monotherapy in various cancers, including gastric, hepatocellular carcinoma, colorectal, and non–small cell lung cancers, resulting in its FDA approval (157–161).

Receptor tyrosine kinase inhibitors offer another promising approach by targeting multiple pathways involved in tumor angiogenesis and growth (Fig. 2A). Unlike VEGFA inhibitors, receptor tyrosine kinase inhibitors act on a broader range of receptors, including VEGFR, FGFR, and platelet-derived growth factor receptor (PDGFR; ref. 162). These drugs have demonstrated clinical benefits in a variety of cancers. For example, sorafenib is FDA approved for renal cell carcinoma, hepatocellular carcinoma, and thyroid cancer, whereas sunitinib has been approved for gastrointestinal stromal tumors, renal cell carcinoma, and pancreatic neuroendocrine tumors (163–166). Similarly, pazopanib has shown efficacy in treating renal cell carcinoma and soft-tissue sarcoma, earning FDA approval (167). Emerging antiangiogenic strategies include targeting the ANG2–TIE2 axis, which plays a key role in vascular stability and remodeling (Fig. 2A). Agents such as MEDI3617, rebastinib, and trebananib are currently under clinical investigation (168). These alternative approaches aim to disrupt angiogenesis more selectively or complement existing therapies. Although significant progress has been made in antiangiogenic therapy, challenges persist. Many agents exhibit limited efficacy in certain cancer types or fail to improve survival when combined with other therapies. Moreover, the heterogeneity of tumor vasculature and resistance mechanisms necessitate further research to refine these strategies. A deeper understanding of tumor biology and the development of combination therapies tailored to specific cancer types hold the key to overcoming these limitations and maximizing the potential of vascular-targeting therapies.

## Vessel Normalization for Drug Delivery

Vascular integrity is crucial for effective drug delivery and therapeutic success (169). Leaky blood vessels within tumors can hinder the delivery of anticancer agents, while poor vascular perfusion increases interstitial fluid pressure, creating physical barriers to drug penetration (170). Additionally, the tumor ECM can collapse microvessels, trapping drugs and further obstructing therapy efficacy (171). Combining antiangiogenic therapies with chemotherapy has been shown to improve vessel function, reduce interstitial fluid pressure, and enhance drug delivery to tumors (172). For example, a phase II clinical trial (NCT00035656) demonstrated that cediranib, a potent VEGFR inhibitor targeting VEGFR-1, VEGFR-2, and VEGFR-3, promoted vessel normalization and improved blood flow in patients with glioblastoma (173). Similarly, bevacizumab, an anti-VEGFA agent, has been shown to enhance tumor blood perfusion, reduce microvascular density, and lower interstitial fluid pressure in patients with colorectal cancer (174). These findings highlight the potential of vessel normalization as a strategy to improve drug delivery and therapeutic outcomes in cancer treatment.

## Vascular-Targeted Therapies in Combination with Immunotherapies

Targeting tumor vasculature in combination with immunotherapies presents a promising strategy to enhance the effectiveness of conventional cancer therapies (169). Neovascularization plays a pivotal role in cancer survival, progression, and metastasis, making the tumor vasculature an attractive therapeutic target (175). Research has shown that blood vessels in glioblastomas, a highly vascularized and immune-resistant solid tumor, express p21-activated kinase 4 (PAK4), an enzyme involved in regulating genetic reprogramming and abnormal vascularization (176). A study revealed that inhibiting PAK4 can reprogram the tumor vasculature and improve the effectiveness of CAR T-cell therapy in glioblastoma (177). Glioblastomas are known for their aberrant vasculature, which creates an immune-suppressive microenvironment that hampers the efficacy of immunotherapies (178). By combining PAK4 inhibition with CAR-T cells engineered to target the EGFR variant III, researchers successfully reprogrammed the vasculature, facilitating immune cell adhesion and improving the penetration of T cells into the tumor. This approach led to a significant anticancer response in preclinical models (177). These findings suggest that targeting PAK4-induced endothelial cell plasticity could be a key strategy to reprogram the TME and boost the success of cancer immunotherapy.

Moreover, many combination therapies have received FDA approval, with many others under investigation (83). Examples include immune checkpoint inhibitors combined with antiangiogenic drugs such as axitinib, cabozantinib, and lenvatinib, which have been approved for the treatment of renal cell carcinoma (82). In addition, adding atezolizumab to bevacizumab and chemotherapy improved survival in metastatic nonsquamous non–small cell lung cancer, and the combination is now approved (179). All in all, combining vascular normalization with immunotherapy is gaining validation in clinical practice.

## Lymphatic Vessels and Lymphangiocrine Signaling in Cancer

The role of tumor-associated LECs and their interaction with lymphangiocrine factors, cancer cells, and stromal cells have been widely studied (Fig. 2B; refs. 180–182). Lymphangiocrine factors are crucial for promoting angiogenesis, increasing vascular permeability in premetastatic sites, and modulating the immune response (Fig. 2B). These activities underscore the significant influence of lymphangiocrine signaling in cancer progression (Supplementary Table S1; ref. 180). LECs facilitate communication with tumor cells through a range of signaling molecules, including interleukins and chemokines like CXCLs and CCLs, which play essential roles in cancer cell migration (Fig. 2B; refs. 183–185). For example, LECs express chemokines such as CXCL12 and CCL21, which attract tumor cells that express the receptors CXCR4 and CCR7, guiding their invasion into lymphatic vessels (185). This chemokine–ligand/receptor interaction suggests that LECs mediate tumor-specific mechanisms, facilitating cancer cell invasion.

In the case of breast cancer metastasis, tumor cells secrete IL6, which activates STAT3 signaling in LECs (183, 186). This activation induces the expression of HIF1α and VEGF, whereas a complex of pSTAT3–pc–Jun–pATF-2 leads to the secretion of CCL5 by LECs. CCL5 attracts cancer cells expressing CCR5 into the lymphatics, promoting lymph node metastasis. Inhibition of IL6 or STAT3 signaling reduces CCL5 secretion by LECs, significantly decreasing lymphatic metastasis (186).

Lymphatic vessels play a vital role in metastasis and immune modulation within the TME. They interact with stromal and immune cells to create an immunosuppressive environment in advanced tumors (187). For example, in cholangiocarcinoma, CAFs interact with LECs through signaling pathways. PDGFD stimulation of CAFs increases VEGFC and VEGFA secretion, which recruits LECs expressing VEGFR2 and VEGFR3, enhancing lymphatic vessel permeability and facilitating cancer cell migration across the lymphatic endothelium (188, 189). This process can be blocked by the PDGFRβ inhibitor imatinib, and CAF depletion in a cholangiocarcinoma rat model reduces lymphatic vascularization and lymph node metastasis (189).

LECs also play an essential role in immune regulation within the lymphatic system. They can suppress T-cell activity by expressing immune checkpoint molecules and by presenting antigens without the necessary co-stimulatory signals, contributing to immune evasion (Fig. 2B; ref. 190). High levels of VEGF-C are associated with increased metastasis and poor prognosis in several cancers. However, in melanoma, high VEGF-C levels and prominent lymphangiogenesis are paradoxically linked to improved responses to immunotherapy, illustrating the complex role of lymphangiocrine signaling in cancer (191).

Aging is a major risk factor for cancer, and emerging research is shedding light on the impact of aging on the lymphatic vascular system and its involvement in tumor progression. In aged tissues, a reduction in matrix complexity, especially in dermal and lymphatic fibroblast matrices, has been observed. For example, decreased production of HAPLN1, a protein responsible for crosslinking hyaluronan to the ECM, leads to increased permeability of peritumoral lymphatic vessels and enhances metastatic spread in aged patients with melanoma and in mouse models (192). Aging also affects lymph nodes, which undergo structural changes that impair immune function and contribute to an increased risk of cancer metastasis (192). These age-related changes may explain the susceptibility of older individuals to cancer spread.

Targeting lymphangiocrine signaling pathways presents a promising avenue for the development of more targeted and effective cancer therapies. Understanding the interactions between lymphatic vessels, immune cells, and stromal components in both aging and cancer could lead to the development of novel therapies that exploit the lymphatic system to enhance antitumor immunity and limit metastatic spread.

## Conclusion and Future Perspectives

This review emphasizes the critical role of the vascular and stromal cells in driving cancer progression, with a particular focus on the stromal components and their influence on metastasis and the EMT. The dynamic interactions between cancer cells and stromal elements, such as CAFs and the ECM, are pivotal in fostering tumor growth, promoting therapeutic resistance, and complicating treatment strategies.

Stromal cells within the TME play an active role in shaping immune-suppressive mechanisms, sustaining EMT, and supporting resistance pathways. These factors underscore their potential as targets for innovative cancer therapies. Recent advancements in TME-targeted approaches have shown promise, particularly in mitigating immune suppression, enhancing anticancer immunity, and improving the precision of immune-targeted drugs. However, significant hurdles remain, including both primary and acquired resistance to these therapies. A deeper understanding of vascular and stromal cell contributions and their interactions with other TME components is essential to overcoming these challenges.

Future efforts must focus on unraveling the specific vulnerabilities of stromal cells and their networks within the TME. Key research priorities include developing strategies to disrupt stromal support for cancer progression, refining patient stratification based on TME characteristics, and exploring novel therapeutic combinations. Additionally, integrating advanced diagnostic tools for early cancer detection will be vital for achieving transformative outcomes in oncology. In conclusion, targeting stromal cells within the TME represents a compelling avenue for advancing cancer therapy. Continued exploration of these elements is crucial for unlocking the full potential of precision oncology, enhancing therapy efficacy, and ultimately improving patient outcomes.

## Supplementary Material

Supplementary Data supplementary tables and text.
