# Supplementary material for: Transforming Cancer Therapy: Unlocking the Potential of Targeting Vascular and Stromal Cells in the Tumor Microenvironment
Source: Cancer Res. 2025 Apr 2;85(12):2152–64. doi: 10.1158/0008-5472.CAN-24-4744 (PMC12167934; doi:10.1158/0008-5472.CAN-24-4744)
Supplement: Supplementary Data — supplementary tables and text. [file can-24-4744_supplementary_data_suppsd.docx]

**Supplementary Tables**

**Supplementary Table S1: The role of vascular and stromal cells in cancer**

| Cell type | Factors / Signal pathway | Cancer type(s) | Role / Impact | Model | PMID |
| --- | --- | --- | --- | --- | --- |
| LECs | CCR7-CCL21 | Non-small cell lung cancer | LEC-secreted CCL21 contributes to the lymphatic metastasis of cancer cells via the CCR7-CCL21 axis. | Patients/in vitro | PMID: 33158173 |
| LECs | CCR7-CCL21 | Breast cancer | TGF-β1 promotes CCL21 expression in LECs;  CCL21 mediates chemotactic migration of EMT cells toward LECs. | Patients/in vitro  Mice | PMID: 25961925 |
| LECs | CXCL12-CXCR4-CCR7 | Breast cancer | CXCL12 expressing LNs recruit CXCR4 and CXCR7 cancer cells, promoting lymphatic metastasis. | Patients/in vitro | PMID: 35133060 |
| LECs | CXCR4/ACKR3/CXCL12 | Vulvar squamous cell carcinoma | LEC-secreted CXCL12, activates CXCR4/ACKR3/CXCL12 axis during progression and lymphatic metastasis. | Patients | PMID: 33692092 |
| LECs | CCL1-CCR8 | Melanoma  Breast cancer | LEC-secreted CCL1 recruits CCR8+ melanoma cells, migrating and promoting lymph node metastasis. | Patients/in vitro Mice | PMID: 23878309 |
| LECs | CCL19-CCR7 | Central nervous system lymphoma  Gastric cancer  Breast cancer  Epithelial ovarian carcinoma | LEC-secreted CCL19 binds on tumour cells and DCs, which facilitate tumor cell migration and metastasis. | Patients/in vitro  Mice | PMID: 35281921  PMID: 28467903  PMID: 31526758  PMID: 37208608 |
| LECs | CXCL1 | Gastric cancer | LEC-secreted CXCL1 promotes the lymphatic metastasis of gastric cancer by activating integrin β1/FAK/AKT signaling. | Mice | PMID: 27832972 |
| LECs | CXCL5-CXCR2 | Cholangiocarcinoma | Inflamed LECs produce CXCL5, promoting metastasis via CXCR2 on cancer cells. | Mice | PMID: 34831316 |
| LECs | EGF-EGFR | Breast cancer | LEC-secreted EGF promotes tumor cell proliferation. | Patients/in vitro Mice | PMID: 25068296 |
| LECs | PDFBB- PDGFRβ | Breast cancer | LEC-secreted PDGF-BB induces pericyte infiltration and angiogenesis. | Patients/in vitro Mice | PMID: 25068296 |
| LECs | IL-17A | Hepatoma | LECs help hepatoma stem cells to self-renew and escape immune attack by upregulating IL-17A signaling. | Patients/in vitro | PMID:31499129 |
| LECs | IL-6 | Skin carcinoma | Tumor exposed-LECs secrete IL-6, promote cancer cell proliferation and invasion. | Patients/in vitro | PMID:33080310 |
| LECs | PD-L1 | Melanoma  Colorectal carcinoma | LECs regulate CD4+ T cell response via PD-L1. | Mice | PMID:34099493 |
| BECs | CXCL12-CXCR4 | Hepatocellular carcinoma | CXCL12+ ECs inhibit CD8+ naïve T cells differentiation into cytotoxic T cells via CXCR4. | Patients/in vitro  Mice | PMID: 39393439 |
| BECs | DGKG | Hepatocellular carcinoma | Endothelial DGKG promotes tumor angiogenesis and immune evasion via the ZEB2/TGF-β1 axis. | Patients/in vitro  Mice | PMID: 37838036 |
| BECs | IL-6-IL-6R | Head and neck squamous cell carcinoma | EC-secreted IL-6 enhances motility and survival of cancer cells. | Patients/in vitro | PMID: 29245982 |
| CAFs | VEGF-A  VEGF-C | Liver cancer | PDGF-D stimulates CAF to secrete VEGF-A and VEGF-C, leading to lymphatic vascularization and tumour cell vascular invasion. | Patients/in vitro  Rats | PMID: 30553841 |
| CAFs | VEGF | Ovarian cancer | CAFs secrete VEGF, supporting angiogenesis, tumor growth, and metastasis. | Patients/in vitro  Mice | PMID: 21911392  PMID: 28978035 |
| CAFs | GDF15  (MIC-1) | Prostate cancer | CAF-derived GDF15 promotes prostate cancer cell migration, invasion, and tumor growth. | Patients/in vitro  Mice | PMID: 24780757 |
| CAFs | TGFβ-fibronectin | Breast cancer | CAFs enhance tumor vascularization by increasing pericyte-endothelium association via TGFβ-fibronectin axis. | Patients/in vitro  Mice | PMID: 29921235 |
| CAFs | TGFβ | Colon cancer  Urinary bladder cancer | CAFs secrete TGFβ to induce EMT;  CAFs induces EMT and invasion of urinary bladder cancer cells through TGFβ1-ZEB2NAT-ZEB2 axis. | Patients/in vitro | PMID: 30835040  PMID: 26152796 |
| CAFs | Hedgehog | Triple negative breast cancer | Hedgehog reprograms CAFs to facilitate cancer chemoresistance through FGF5 and fibrillar collagen. | Patients/in vivo  Mice | PMID: 30042390 |
| CAFs | Notch | Hepatocellular carcinoma | CAF‐induced Notch3 expression leads to LSD1 deacetylation and activation, thus maintaining the self‐renewal and tumorigenicity of cancer stem-like cells. | Mice | PMID: 29259010 |
| CAFs | Notch | Breast cancer | CAF-secreted miR‐221 decreases ER expression and upregulates Notch3 in recipient cancer cells, ultimately promoting hormone therapy resistance. | Patients/in vitro  Mice | PMID: 28202520 |
| CAFs | NF-κB | Squamous skin carcinogenesis | CAFs enhance tumor growth by mediating innate immune cell recruitment, promoting tumor angiogenesis via NF-κB signal. | Patients/in vitro  Mice | PMID: 20138012 |
| CAFs | Wnt | Colorectal cancer | CAFs-derived WNT2 increases tumor angiogenesis and invasion. | Patients  Mice | PMID: 31667643 |
| CAFs | Wnt/β-catenin | Oesophageal squamous cell carcinoma | Wnt2 enhances cell motility and invasiveness by inducing EMT. | Patients/in vitro  Mice | PMID: 21672941 |
| CAFs | IL-6 | Bladder cancer | CAF-secreted IL-6 promotes EMT and tumor aggression. | Patients/in vitro | PMID: 30744595 |
| CAFs | IL-6/STAT3 | Lung cancer | CAFs induce EMT and promote metastasis of lung cancer cells through the IL-6/STAT3 signal. | Patients/in vitro  Mice | PMID: 29100297 |
| CAFs | IL-17a/JAK2/STAT3 | Gastric cancer | CAFs-secreted IL‐17a promotes the migration and invasion of gastric cancer cells. | Patients/in vitro | PMID: 32793721 |
| CAFs | IL-11 | Gastric cancer | CAFs-secreted IL-11 enhances migration and invasion via JAK/STAT3, MAPK/ERK pathways. | Patients/in vitro  Mice | PMID: 29709516 |
| CAFs | IL-8 | Gastric cancer | CAFs derived IL-8 promotes chemoresistance via NF-κB activation and ABCB1. | Patients/in vitro | PMID: 30978440 |
| CAFs | MAPK | Lung cancer | VCAM-1 secreted from CAFs enhances growth and invasion by AKT and MAPK signaling. | Patients/in vitro  Mice | PMID: 31904479 |
| CAFs | PI3K/AKT | Gallbladder cancer | CAFs derived TSP-4 promotes cancer progression via TSP-4/integrin α2/HSF1/TGF-β cascade axis. | Patients/in vitro  Mice | PMID: 33407730 |
| CAFs | Growth factor such as EGF, HGF, TGFβ, and FGF-2 | Endometrial cancer  Ovarian cancer | CAFs secrete growth factors to induce EMT. | Patients/in vitro | PMID: 29563996  PMID: 36424360  PMID: 30710055 |
| CAFs | HGF | MET-unamplified gastric cancer | CAFs promote tumor progression through HGF/c-Met/STAT3/twist1, and (IL)-6/IL-6R/JAK2/STAT3/twist1 pathway. | Patients/in vitro  Mice | PMID: 30158543  PMID: 36424360 |
| CAFs | IGF2-IGF1R-YAP1 | Colorectal cancer | CAFs promote cancer progression via IGF2-IGF1R-YAP1 signaling. | Patients/in vitro  Mice | PMID: 36373776 |
| CAFs | YAP | Breast cancer | YAP is required for pro-tumorigenic functions of CAFs, including matrix stiffening, invasion, and angiogenesis. | Patients/in vitro  Mice | PMID: 23708000 |
| CAFs | MRTF–SRF  YAP–TEAD | Mammary tumor | MRTF–SRF and YAP–TEAD signaling are required for CAF’s contractile and proinvasive properties. | Patients  Mice | PMID: 29317486 |
| CAFs | LOX | Liver metastasis of gastric cancer | CAFs secrete LOX to support tumor cells in liver metastatic niche of gastric cancer. | Patients/in vitro  Mice | PMID: 31678002 |
| CAFs | LOXL2 | Prostate cancer | CAF-derived LOXL2 promotes CAF motility and ECM modelling. | Patients/in vitro | PMID: 31061140 |

**Supplementary Table S2: Roles of CAF subtypes in cancer**

| CAF Type | Cancer Type(s) | Function | Markers | Model | PMID |
| --- | --- | --- | --- | --- | --- |
| Myofibroblastic CAFs  (myCAFs) | Pancreatic ductal adenocarcinoma | ECM production | αSMA, CTGF, TNC, TAGLN;  αSMA, TAGLN, LRCC15 | Patients  Mice | PMID: 30366930  PMID: 31197017  PMID: 30385653  PMID: 31335328  PMID: 31699795 |
| Myofibroblastic CAFs  (myCAFs) | Head and neck cancer | Contractile activity | αSMA^High^, MYL9, MYLK | Patients | PMID: 29198524 |
| Myofibroblastic CAFs  (myCAFs) | Intrahepatic cholangiocarcinoma | Tumor promotion | APOD, CCL11, COL1A1, COL1A2, COL3A1, COL5A1, COL6A3, CTGF, CTHRC1, CYP1B1, FN1, INHBA, ISLR, LUM, MMP14, POSTN, PTGDS, SERPINF1, SFRP2, SPON2, VCAN;  Acta2, Col12a1, Col15a1, Col1a1, Col1a2, Col3a1, Cola4a5, Col5a2, Col5a3, Col6a2, Col7a1, Col8a1, Colec12, Fbln1, Fbln2, Fbn2, Fgfr1, Heyl, Igfbp5, Inhba, Lama4, Lxn, Mmp14, Mmp2, Mtch1, Nb11, Ncam1, Nduga412, Nkd2, Pdgfrl, Plat, Plaur, Ptn, Runx1, S100a16, S100a4, Serpine2, Serpinf1, Ssp1, Thy1, Tnc, Vcan, Vegfa | Patients  Mice | PMID: 33930309 |
| Myofibroblastic CAFs  (myCAFs) | Lung adenocarcinoma  Colon adenocarcinoma  Breast cancer  Cholangiocarcinoma  Stomach cancer  Neuroendocrine prostate cancer  Pancreatic ductal adenocarcinoma  Uterine corpus endometrial carcinoma  Ovarian cancer  Uveal melanoma | Contractile activity | RGS5, MYH11, ACTA2 | Patients | PMID: 38148640 |
| Myofibroblastic CAFs  (myCAFs) | Breast cancer | Contractile activity | αSMA, MYLK | Mice | PMID: 33852842 |
| Myofibroblastic CAFs  (myCAFs) | Colorectal cancer | ECM remodeling | RGS5 | Patients | PMID: 35794563 |
| Myofibroblastic CAFs  (myCAFs) | Basal cell carcinoma  Squamous cell carcinoma  Melanoma | Not clear | RGS5 | Patients | PMID: 39516494 |
| Inflammatory CAFs  (iCAFs) | Pancreatic ductal adenocarcinoma | Immunosuppression/ Tumor promotion | Ly6C^High^, αSMA^Low^, CXCL12, PDGFRα^High^, C3, IL6 | Patients  Mice | PMID: 30366930  PMID: 31197017  PMID: 30385653  PMID: 31335328  PMID: 31699795 |
| Inflammatory CAFs  (iCAFs) | Intrahepatic cholangiocarcinoma | Tumor promotion | ADAMTS4, AGT, APOE, ARHGDIB, CCL19, CCL21, COLEC11, CPE, GEM, GJA4, GPX3, HIGD1B, IL-6, ISYNA1, LHFP, MAP1B, MT1A, NDUFA4L2, PDK4, RGS5;  CXCL1, C3, C7, FBLN1, IGFI, IGFBP6, SAA1 | Patients  Mice | PMID: 37018128  PMID: 33930309  PMID: 32505533 |
| Inflammatory CAFs  (iCAFs) | Breast cancer | Immunomodulation | Ly6C^High^, C3, CXCL12, PDGFRα^High^ | Mice | PMID: 33852842 |
| Inflammatory CAFs  (iCAFs) | Lung adenocarcinoma  Colon adenocarcinoma Breast cancer  Cholangiocarcinoma  Stomach cancer  Neuroendocrine prostate cancer  Pancreatic ductal adenocarcinoma  Uterine corpus endometrial carcinoma  Ovarian cancer  Uveal melanoma | Immunomodulation | CCL2, CXCL12, CXCL14 | Patients | PMID: 38148640 |
| Inflammatory CAFs  (iCAFs) | Colorectal cancer | Immunomodulation  EMT  Cholesterol homeostasis  Bile acid metabolism  Fatty acid metabolism | PDGFRA | Patients | PMID: 35794563 |
| Inflammatory CAFs  (iCAFs) | Basal cell carcinoma  Squamous cell carcinoma  Melanoma | Aid immune cell recruitment and activation | TGFB3, LGALS9, IL1B, IL6, CXCR2 ligands, IDO1 | Patients | PMID: 39516494 |
| Antigen-presenting CAFs  (apCAFs) | Pancreatic ductal adenocarcinoma | Immunomodulation (leukocyte cell-cell adhesion, response to IFN-γ, antigen processing, antigen presentation) | MHCII+ | Patients  Mice | PMID: 30366930  PMID: 31197017  PMID: 32505533 |
| Antigen-presenting CAFs  (apCAFs) | Intrahepatic cholangiocarcinoma | Immunomodulation | CD74, HLA-DRA, HLA-DRB1, CCL21, CXCL12;  IGFBP3, CXCL12, HLA-DRB1, CD74, HLA-DRA, RBP1, HLA-DPB1, COLEC11, TMEM56, CCL2 | Patients | PMID: 32505533  PMID: 35584893 |
| Mesothelial CAFs  (mesCAFs) | Intrahepatic cholangiocarcinoma | Not clear | ANXA1, ANXA2, BDKRB1, C19orf33, C3, CALB2, CCDC80, CFB, CRABP2, CXCL1, CXCL6, EFEMP1, EGFL6, EMP3, EZR, HMOX1, HP, HSPA6, IFI27, IGFBP6, ITLN1, KRT18, KRT19, KRT8, LINC01133, LOX, MT1E, MT1G, MT1X, MXRA5, PDPN, PLA2G2A, PRG4, PRSS23, PTGIS, RP11-572C15.6, S100A10, S100A16, S100A6, SAA1, SAA2, SERPINE2, SH3BGRL3, SLC12A8, SLPI, TM4SF1 | Patients  Mice | PMID: 33930309 |
| Vascular CAFs  (vCAFs) | Intrahepatic cholangiocarcinoma | Muscle contraction  Hypoxia response  Mesenchymal cell proliferation | CCL8, GJA4, MHY11, CD146 (MCAM), RGS5, IL-6;  ADIRF, RGS5, SPARCL1, CRIP1, NDUFA4L2, MYH11, MCAM, PDK4, FABP4, TINAGL1 | Patients | PMID: 37018128  PMID: 32505533  PMID: 35584893 |
| Vascular CAFs  (vCAFs) | Breast cancer | Angiogenesis | αSMA^High^ PDGFRβ^High^ | Mice | PMID: 30514914 |
| Epithelial-to-mesenchymal-like CAFs  (eCAFs) | Intrahepatic cholangiocarcinoma | Not clear | KRT19, KRT8, SAA1, SLPI | Patients | PMID: 37018128  PMID: 32505533 |
| Lipofibroblast CAFs  (lCAFs) | Intrahepatic cholangiocarcinoma | Lipid metabolism | APOA2, FABP1, FABP4, FRZB, GPX3 | Patients | PMID: 37018128  PMID: 32505533 |
| Progenitor CAFs  (proCAFs) | Lung adenocarcinoma  Colon adenocarcinoma  Breast cancer  Cholangiocarcinoma  Stomach cancer  Neuroendocrine prostate cancer  Pancreatic ductal adenocarcinoma  Uterine corpus endometrial carcinoma  Ovarian cancer  Uveal melanoma | Differentiate into CAFs | IGF1, OGN, C7 | Patients | PMID: 38148640 |
| Matrix CAFs  (mCAFs) | Cholangiocarcinoma  Stomach cancer  Neuroendocrine prostate cancer  Pancreatic ductal adenocarcinoma  Uterine corpus endometrial carcinoma  Ovarian cancer  Uveal melanoma | ECM reformation | COL10A1, CTHRC1, POSTN | Patients | PMID: 38148640 |
| Matrix CAFs  (mCAFs) | Breast cancer | ECM producing | αSMA^Low^ PDGFRα^High^ | Mice | PMID: 30514914 |
| Matrix CAFs  (mCAFs) | Intrahepatic cholangiocarcinoma | Not clear | COL5A1, COL5A2, COL6A3, DCN, FN1, LUM, POSTN, VCAN;  POSTN, CTHRC1, COL1A1, COL6A3, FN1, COL3A1, MMP14, LUM, SPON2, COL5A1 | Patients | PMID: 32505533  PMID: 35584893 |
| Matrix CAFs  (mCAFs) | Basal cell carcinoma  Squamous cell carcinoma  Melanoma | ECM producing,  Restrict T cell invasion in low-grade tumor | COL11A1, PTGDS,  ACTA2^Low^ | Patients | PMID: 39516494 |
| Inflammatory/myofibroblastic CAFs  (imCAFs) | Intrahepatic cholangiocarcinoma | Immunomodulation | CCDC80, C3, SERPINF1, PTGDS, FBLN1, IGF1, CRABP2, MMP2, CTGF | Patients | PMID: 37018128  PMID: 35584893 |
| Cycling CAFs  (cCAFs) | Breast cancer | Angiogenesis | PDGFRβ | Mice | PMID: 30514914 |
| Developmental CAFs  (dCAFs) | Breast cancer | Cell differentiation | PDGFRβ-, SOX9+, SCRG1+ | Mice | PMID: 30514914 |
| ECM-CAFs | Breast cancer | ECM production | TNC | Mice | PMID: 33852842 |

**Supplementary Table S3:** **Clinical trials of vascular and stromal cell targeting therapy**

| Cell Type | Agent | Target | Mechanism | Cancer type(s) | Status | PMID |
| --- | --- | --- | --- | --- | --- | --- |
| CAFs | RO6874281 | FAP | Interferes CAF function  Promotes T-cell responses | Advanced solid tumors | Phase II | PMID: 38630781 |
| CAFs | Sibrotuzumab | FAP | Interferes CAF function  Promotes T-cell responses | Metastatic colorectal cancer | Phase II | PMID: 12624517 |
| CAFs | 177Lu-LNC1004  177Lu-DOTA-EB-FAPI | FAP | Interferes CAF function  Promotes T-cell responses | Recurrent or metastatic FAP+ solid tumors | Phase I/II | PMID: 39238004 |
| CAFs | 177Lu-FAP-2286 | FAP | Interferes CAF function  Promotes T-cell responses | Pancreatic ductal adenocarcinoma  Non-small cell lung cancer  Breast cancer | Phase I/II | PMID: 39238004 |
| CAFs | Galunisertib | TGFβ | Prevents CAF activation  Interferes CAF signaling | Colorectal cancer | Phase II | PMID: 35952709 |
| CAFs | Luspatercept | TGF-β | Prevents CAF activation  Interferes CAF signaling | Myelodysplastic syndromes | Phase III | PMID: 31914241 |
| CAFs | Belagenpumatucel-L | TGF-β | Prevents CAF activation  Interferes CAF signaling | Non-small cell lung cancer | Phase III | PMID: 26283035 |
| CAFs | Erdafitinib | FGFR | Prevents CAF activation | Advanced urothelial carcinoma | Phase III | PMID: 36186154 |
| CAFs | Axitinib | PDGFR | Interferes CAF recruitment | Cytokine-refractory metastatic renal-cell cancer | Phase II | PMID: 17959415 |
| CAFs | Plerixafor | CXCR4 antagonist | Interferes CAF signaling | Multiple myeloma  Non-Hodgkin's lymphoma | Phase III | PMID: 19363221  PMID: 19720922 |
| CAFs | Saridegib | Hedgehog | Prevents and reduces CAF activation | Recurrent/metastatic head and neck squamous cell carcinoma | Phase I | PMID: 31035664  PMID: 25646180  PMID: 26705064 |
| CAFs | Vismodegib | Hedgehog | Prevents and reduces CAF activation | Basal cell carcinoma | FDA approved | PMID: 25981813  PMID: 39001766 |
| CAFs | Sonidegib | Hedgehog | Prevents and reduces CAF activation | Basal cell carcinoma | FDA approved | PMID: 31545507 |
| CAFs | AT13148 | ROCK | Interferes CAF function | Solid tumors | Phase II | PMID: 32616501 |
| CAFs | ATRA | Vitamin A | CAF normalization | Adenoid cystic carcinoma  Acute promyelocytic leukemia | Phase II | PMID: 34091189  PMID: 27400939 |
| CAFs | Paricalcitol | Vitamin D | CAF normalization | Pancreatic cancer | Phase II | PMID: 36299300 |
| CAFs | Tranilast | IL-6 | Inhibits CAFs from secreting IL-6  Suppresses upregulation of p-STAT3  Inhibits EMT in NSCLC cells | Nasopharyngeal carcinoma | Phase II | PMID: 35871750  PMID: 34174504  PMID: 38602644 |
| CAFs | Pirfenidone | Type I collagen | Reduces type I collagen expression  Enhances radiosensitivity | Head and neck squamous cell carcinoma | Phase II | PMID: 26935219  PMID: 32616870  PMID: 37968249 |
| BECs | Cediranib | VEGF/VEGFR | Antiangiogenesis | Advanced solid tumors | FDA approved | PMID: 38231047 |
| BECs | Aflibercept | VEGF/VEGFR | Antiangiogenesis | Advanced solid tumors | FDA approved | PMID: 24368879 |
| BECs | Ramucirumab | VEGF/VEGFR | Antiangiogenesis | Advanced solid tumors | FDA approved | PMID: 24094768 |
| BECs | WX-671 | uPAR | Antiangiogenesis | Breast cancer | Phase II | PMID: 35158766 |
| BECs | Axitinib | RTKs | Antiangiogenesis | Advanced solid tumors | Phase III | PMID: 23598172 |
| BECs | Sorafenib | RTKs | Antiangiogenesis | Advanced solid tumors | FDA approved | PMID: 17215530 |
| BECs | Sunitinib | RTKs | Antiangiogenesis | Advanced solid tumors | FDA approved | PMID: 27836885 |
| BECs | Pazopanib | RTKs | Antiangiogenesis | Advanced solid tumors | FDA approved | PMID: 23964934 |
| BECs | MEDI3617 | ANG2–TIE2 | Antiangiogenesis | Advanced solid tumors | Phase I | PMID: 29559563 |
| BECs | Rebastinib | ANG2–TIE2 | Antiangiogenesis | Advanced solid tumors | Phase II | PMID: 34440616 |
| BECs | Trebananib | ANG2–TIE2 | Antiangiogenesis | Advanced solid tumors | Phase III | PMID: 31076365 |
| BECs | Lenvatinib | RTKs | Antiangiogenesis | Leiomyosarcoma  Undifferentiated pleomorphic sarcoma  Vascular sarcomas  Synovial sarcoma or malignant peripheral nerve sheath tumor  Bone sarcomas | Phase II | PMID: 39405335 |
| BECs | Lenvatinib | RTKs | Metastatic endometrial cancer | Advanced endometrial carcinoma | Phase II | PMID: 30922731 |
| BECs | Bevacizumab | VEGF-A | Antiangiogenesis | Non-squamous non-small-cell lung cancer | Phase III | PMID: 29863955 |
| BECs | Bevacizumab | VEGF-A | Antiangiogenesis | Metastatic colorectal cancer | Phase III | PMID: 38565886 |
| BECs | Bevacizumab | VEGF-A | Antiangiogenesis  Immunomodulatory | Untreated locally advanced or metastatic HCC | Phase III | PMID: 32402160 |
| BECs | Axitinib  Sunitinib | RTKs  VEGF | Antiangiogenesis  Immunomodulation | Advanced renal-cell carcinoma | Phase III | PMID: 30779529 |
| BECs | Axitinib  Sunitinib | VEGFR | Antiangiogenesis  Immunomodulation | Advanced renal-cell carcinoma | Phase III | PMID: 30779531 |
| LECs | VGX-100 | VEGF-C | Targets VEGF-C to inhibit VEGFR-2 and VEGFR-3 activation | Advanced solid tumors | Phase I | PMID: 35771983 |
| LECs | IMC-3C5 | VEGFR-3 | Inhibits tumor lymphangiogenesis | Advanced refractory solid tumors  Advanced colorectal cancer | Phase I | PMID: 27566701 |
| LECs | Anlotinib | RTKs | Inactivates VEGFR-3 phosphorylation | Lung adenocarcinoma | Phase III | PMID: 32944404 |
| LECs | Sunitinib | RTKs | Inhibits VEGFR and other receptors | Breast cancer | Phase II | PMID: 34928481 |
| Hyaluronan | PEGPH20 | Hyaluronidase | Degrades ECM | Previously untreated pancreatic ductal adenocarcinoma | Phase IB/II | PMID: 26813359 |
| Hyaluronan | PEGPH20 | Hyaluronidase | Degrades ECM | Metastatic pancreatic ductal adenocarcinoma | Phase II | PMID: 30614815 |
| Hyaluronan | 4-MU | Hyaluronan synthesis | Degrades ECM | Pancreatic cancer  Lung cancer | Phase I | PMID: 35499083 |
| Fibronectin/integrins | VS-4718 (PND-1186) | FAK | Reduces ECM stiffness  Inhibits FAK1/PTK2 and Pyk2/PTK2b kinases | Metastatic nonhematologic cancers | Phase I | PMID: 27262114  PMID：20234191 |
| Fibronectin/integrins | VS-4718 (PND-1186) | FAK | Reduces ECM stiffness  Inhibits FAK1/PTK2 and Pyk2/PTK2b kinases | Pancreatic cancer | Phase I | PMID: 27262114 |
| Fibronectin/integrins | VS-4718 (PND-1186) | FAK | Reduces ECM stiffness  Inhibits FAK1/PTK2 and Pyk2/PTK2b kinases | Acute myeloid or B-cell acute lymphoblastic leukemia | Phase I | PMID: 27262114 |
| Fibronectin/integrins | VS-6062 (PF-562271, PF-271) | FAK | Reduces ECM stiffness  Reversibly inhibits FAK/Pyk2 | Head and neck cancer  Prostate cancer  Pancreatic cancer | Phase I | PMID: 22454420 |
| Fibronectin/integrins | Defactinib (VS-6063, PF-04554878) | FAK | Reduces ECM stiffness  Inhibits FAK/Pyk2 | Ovarian serous tumors | Phase IB/II | PMID: 35929603 |
| Fibronectin/integrins | Defactinib (VS-6063, PF-04554878) | FAK | Reduces ECM stiffness  Inhibits FAK/Pyk2 | Pancreatic ductal adenocarcinoma | Phase I | PMID: 36228156 |
| Fibronectin/integrins | Defactinib (VS-6063, PF-04554878) | FAK | Reduces ECM stiffness  Inhibits FAK/Pyk2 | Merlin-stratified pleural mesothelioma | Phase II | PMID: 30785827 |
| Fibronectin/integrins | Defactinib (VS-6063, PF-04554878) | FAK | Reduces ECM stiffness  Inhibits FAK/Pyk2 | KRAS mutant non-small cell lung cancer | Phase II | PMID: 31739184 |
| Fibronectin/integrins | Defactinib (VS-6063, PF-04554878) | FAK | Reduces ECM stiffness  Inhibits FAK/Pyk2 | Intractable pancreatic cancer | Phase I | PMID: 36228156 |
| Fibronectin/integrins | Defactinib (VS-6063, PF-04554878) | FAK | Reduces ECM stiffness  Inhibits FAK/Pyk2 | KRAS mutant non-small cell lung cancer | Phase II | PMID: 35285277 |
| Fibronectin/integrins | Defactinib (VS-6063, PF-04554878) | FAK | Reduces ECM stiffness  Inhibits FAK/Pyk2 | Nonhematologic cancers | Phase I | PMID: 27262114 |
| Fibronectin/integrins | Defactinib (VS-6063, PF-04554878) | FAK | Reduces ECM stiffness  Inhibits FAK/Pyk2 | Advanced nonhematologic cancers | Phase I | PMID: 27262114 |
| Fibronectin/integrins | Defactinib (VS-6063, PF-04554878) | FAK | Reduces ECM stiffness  Inhibits FAK/Pyk2 | Advanced malignant neoplasm  Lymphoma  Refractory malignant neoplasm  Solid neoplasm | Phase II | PMID: 27262114 |
| Fibronectin/integrins | IN10018 (BI853520) | FAK | Reversibly inhibits FAK/Pyk2 | Advanced or metastatic cancer | Phase I | PMID: 27262114 |
| Fibronectin/integrins | IN10018 (BI853520) | FAK | Reduces ECM stiffness | Metastatic non-hematologic malignancy  Advanced or metastatic solid tumors  Platinum-resistant ovarian cancer | Phase I/II | PMID: 30756308  PMID: 30725402  PMID: 35598365 |
| Fibronectin/integrins | Conteltinib | FAK | Reduces ECM stiffness | ALK-positive non-small cell lung cancer | Phase I | PMID: 36424628 |
| Fibronectin/integrins | GSK-2256098 | FAK | Reduces ECM stiffness | Advanced solid tumors | Phase I/IB | PMID: 27733373  PMID: 30992546 |
| Fibronectin/integrins | GSK-2256098 | FAK | Reduces ECM stiffness | NF2 mutant meningioma | Phase II | PMID: 36288512 |
| Fibronectin/integrins | GSK-2256098 | FAK | Reduces ECM stiffness | Solid tumors | Phase I | PMID: 27262114 |
| Fibronectin/integrins | GSK-2256098 | FAK | Reduces ECM stiffness | Idiopathic pulmonary arterial hypertension | Phase I | PMID: 27262114 |
| Fibronectin/integrins | GSK-2256098 | FAK | Reduces ECM stiffness | Advanced pancreatic cancer | Phase II | PMID: 27262114 |
| Fibronectin/integrins | VS-6766 (RO5126766) | FAK | Inhibits RAF/MEK | Non-small-cell lung cancer  Low-grade serous ovarian cancer  Endometrioid cancer  Pancreatic cancer | Phase I | PMID: 35929603 |
| Fibronectin/integrins | VS-6766 (RO5126766) | FAK | Inhibits RAF/MEK | Ovarian cancer | Phase II | PMID: 35929603 |
| Fibronectin/integrins | VS-6766 (RO5126766) | FAK | Inhibits RAF/MEK | Non-small-cell lung cancer (KRAS G12V, or other KRAS or BRAF mutations) | Phase II | PMID: 35929603 |
| Fibronectin/integrins | APG-2449 | FAK/PYK2/ALK | Reduces ECM stiffness | Advanced solid tumor | Phase IB/II | PMID: 38410129 |
| Fibronectin/integrins | ARV-110  ARV-471 | PROTACs (Proteolysis targeting chimeras) | Reduces ECM stiffness through a kinase-independent way | Metastatic castration resistant prostate cancer  Locally advanced or metastatic breast cancer | Phase I | PMID: 32404196 |

**Supplementary Text**

**Targeting ECM-degrading proteinases**

MMP inhibitors, including AZ11557272, AS111793, MMP-408, and AZD1236, have shown potential in preclinical studies and early clinical trials (1). GM6001, a potent MMP inhibitor, demonstrates antimetastatic effects (2), while BAY 12-9566N reduces tumor growth and progression (3). However, phase III clinical trials of MMP inhibitors have failed due to poor specificity, off-target effects like musculoskeletal syndrome, metabolic instability, and dose-limiting toxicities (1).

Cathepsin inhibitors have been explored in cancer therapy. Preclinical and gene ablation studies suggest that cathepsin inhibition delays tumor growth but does not eliminate tumors, limiting their effectiveness as standalone therapies (4). Reversible cathepsin B inhibitors, like nitroquinoline and its derivatives, reduce ECM degradation and hinder tumor progression (5,6). The cathepsin B inhibitor VBY-825 shows significant tumor suppression in pancreatic cancer models by decreasing proliferation and increasing apoptosis but it is not tested further (7). Selective inhibitors of cathepsin S, such as VBY-999, reduce tumor burden during early-stage brain metastasis but are ineffective at later stages (8).

# **Roles of endothelial progenitor cells (EPCs) and circulating endothelial cells (CECs) in cancer progression and therapy**

## ***EPCs in tumor vascularization***

Tumor vasculature develops through endothelial cell sprouting and postnatal vasculogenesis, where bone marrow-derived EPCs migrate to sites of neovascularization and differentiate into endothelial cells (9) (Figure 1B). As reservoirs for vascular repair, EPCs synergize with angiogenesis, exhibiting traits like proliferation, migration, and differentiation, similar to embryonic angioblasts. Elevated EPC levels in cancer patients correlate with disease progression, driven by bone marrow-derived signals that support tumor vascularization. VEGF regulates EPC mobilization and homing via VEGFR-1 and VEGFR-2, with elevated VEGF levels releasing EPCs from the bone marrow within 24 hours (10,11). Additional stimuli, such as cytokines and hormonal factors, further enhance EPC recruitment, while TNF-α and CRP impair their function through apoptosis (12). EPC contributions vary by tumor grade, histology, and therapy, with poorly differentiated tumors attracting more EPC integration (13-15). Beyond structural incorporation, EPCs secrete angiogenic cytokines, emphasizing their dual role in tumor angiogenesis (16).

Emerging evidence suggests that EPCs also play a critical role in shaping the tumor microenvironment, acting as key mediators of crosstalk between stromal and immune cells to promote angiogenesis and tumor progression. EPCs secrete a variety of pro-angiogenic factors, including SDF-1 and MMPs, which facilitate ECM remodeling and create a permissive niche for vascular development. By enhancing effector immune cell infiltration and reducing vascular-mediated immune barriers, these approaches aim to improve the efficacy of anti-cancer therapies, addressing the challenges posed by immune-resistant tumors (17). Furthermore, EPCs interact with perivascular cells, such as pericytes and smooth muscle cells, to stabilize nascent vessels, thereby contributing to the maturation and functionality of tumor vasculature. Pericytes regulate microcirculation and play a key role in tumor progression by promoting angiogenesis, metastasis, and drug resistance through paracrine signaling and interactions with immune cells. Changes in the TME, such as pericyte detachment, further facilitate tumor cell invasion, capillary remodeling, and metastatic spread (18).

## ***CECs as biomarkers for tumor vascularization***

CECs, mature endothelial cells shed into the bloodstream during vascular remodeling or damage, are emerging as biomarkers for tumor vascularization (Figure 1A). CECs are not to be confused with EPCs which originate from bone marrow and are recruited to sites of vascular injury to undergo repair (19). The utility of CECs as a cell-based biomarker for vascular dysfunction has been demonstrated across various diseases, including cardiovascular disease, COVID-19, metabolic disorders, preeclampsia, and sickle cell anemia (20-22). In tumors, CECs may arise from both the vasculature supporting tumor growth and normal vessels undergoing systemic responses to the disease. While CEC levels are low under normal conditions, they increase significantly in pathological states like tumor-induced neovascularization (23). Elevated CEC levels correlate with malignancies such as multiple myeloma, metastatic carcinoma, and myelodysplastic syndrome, tracking disease progression and therapy response (24). Effective chemotherapy often reduces CEC levels, reinforcing their potential as cancer biomarkers. The integration of CEC monitoring into clinical practice could revolutionize cancer management by enabling real-time assessment of tumor vascularization and therapeutic responses (25). Ongoing research aims to refine the specificity of CEC-based assays, enhance the understanding of their molecular signatures, and explore their utility in combination with other liquid biopsy tools, such as circulating tumor cells and extracellular vesicles. Beyond tumor-specific roles, CECs reflect systemic endothelial activation or damage, making them valuable in assessing vascular health, staging diseases, and evaluating therapies.

## ***Therapeutic targeting of EPCs and CECs in cancer***

EPCs play critical roles in tumor resistance to vascular-targeting therapies, such as vascular-disrupting agents (VDAs) and anti-angiogenic drugs. VDAs mobilize EPCs from the bone marrow, promoting tumor vessel regrowth post-treatment. Combining VDAs with VEGFR2 inhibitors or genetic modifications suppresses EPC mobilization and enhances efficacy (26). Similarly, EPC mobilization contributes to chemotherapy resistance, as taxanes—unlike gemcitabine—trigger EPC release at high doses. Combining paclitaxel with VEGFR2 inhibitors reduces this mobilization, while metronomic chemotherapy suppresses EPC activity, enhancing anti-angiogenic effects (27,28). However, anti-angiogenic drugs may induce hypoxia and activate HIF-1α, promoting EPC recruitment via VEGF and SDF-1α, complicating therapy outcomes (29,30).

Beyond resistance mechanisms, EPCs and CECs also serve as therapeutic tools. Ex vivo-modified EPCs and CECs deliver therapeutic agents directly to tumors. For instance, HSV-TK-expressing EPCs with ganciclovir significantly reduced tumors in mice without systemic toxicity (31), while IL-2-expressing CECs eradicated melanoma metastases (32). These findings highlight their dual potential as therapeutic targets and delivery vehicles in cancer therapy.

# **References**

1. Agraval H, Kandhari K, Yadav UCS. MMPs as potential molecular targets in epithelial-to-mesenchymal transition driven COPD progression. Life Sci **2024**;352:122874-

2. Almholt K, Juncker-Jensen A, Laerum OD, Dano K, Johnsen M, Lund LR*, et al.* Metastasis is strongly reduced by the matrix metalloproteinase inhibitor Galardin in the MMTV-PymT transgenic breast cancer model. Mol Cancer Ther **2008**;7:2758-67

3. Iatropoulos MJ, Cerven DR, de George G, von Keutz E, Williams GM. Reduction by dietary matrix metalloproteinase inhibitor BAY 12-9566N of neoplastic development induced by diethylnitrosamine, N-nitrosodimethylamine, or 7,12-dimethylbenz(a)anthracene in rats. Drug Chem Toxicol **2008**;31:305-16

4. Rot AE, Hrovatin M, Bokalj B, Lavrih E, Turk B. Cysteine cathepsins: From diagnosis to targeted therapy of cancer. Biochimie **2024**;226:10-28

5. Mirkovic B, Renko M, Turk S, Sosic I, Jevnikar Z, Obermajer N*, et al.* Novel mechanism of cathepsin B inhibition by antibiotic nitroxoline and related compounds. ChemMedChem **2011**;6:1351-6

6. Sosic I, Mirkovic B, Arenz K, Stefane B, Kos J, Gobec S. Development of new cathepsin B inhibitors: combining bioisosteric replacements and structure-based design to explore the structure-activity relationships of nitroxoline derivatives. J Med Chem **2013**;56:521-33

7. Elie BT, Gocheva V, Shree T, Dalrymple SA, Holsinger LJ, Joyce JA. Identification and pre-clinical testing of a reversible cathepsin protease inhibitor reveals anti-tumor efficacy in a pancreatic cancer model. Biochimie **2010**;92:1618-24

8. Sevenich L, Bowman RL, Mason SD, Quail DF, Rapaport F, Elie BT*, et al.* Analysis of tumour- and stroma-supplied proteolytic networks reveals a brain-metastasis-promoting role for cathepsin S. Nat Cell Biol **2014**;16:876-88

9. Asahara T, Kawamoto A. Endothelial progenitor cells for postnatal vasculogenesis. Am J Physiol Cell Physiol **2004**;287:C572-9

10. Khakoo AY, Finkel T. Endothelial progenitor cells. Annu Rev Med **2005**;56:79-101

11. Asahara T, Takahashi T, Masuda H, Kalka C, Chen D, Iwaguro H*, et al.* VEGF contributes to postnatal neovascularization by mobilizing bone marrow-derived endothelial progenitor cells. EMBO J **1999**;18:3964-72

12. Tousoulis D, Andreou I, Antoniades C, Tentolouris C, Stefanadis C. Role of inflammation and oxidative stress in endothelial progenitor cell function and mobilization: therapeutic implications for cardiovascular diseases. Atherosclerosis **2008**;201:236-47

13. Ruzinova MB, Schoer RA, Gerald W, Egan JE, Pandolfi PP, Rafii S*, et al.* Effect of angiogenesis inhibition by Id loss and the contribution of bone-marrow-derived endothelial cells in spontaneous murine tumors. Cancer Cell **2003**;4:277-89

14. Browder T, Butterfield CE, Kraling BM, Shi B, Marshall B, O'Reilly MS*, et al.* Antiangiogenic scheduling of chemotherapy improves efficacy against experimental drug-resistant cancer. Cancer Res **2000**;60:1878-86

15. Gao D, Nolan DJ, Mellick AS, Bambino K, McDonnell K, Mittal V. Endothelial progenitor cells control the angiogenic switch in mouse lung metastasis. Science **2008**;319:195-8

16. Peters BA, Diaz LA, Polyak K, Meszler L, Romans K, Guinan EC*, et al.* Contribution of bone marrow-derived endothelial cells to human tumor vasculature. Nat Med **2005**;11:261-2

17. Kim HJ, Ji YR, Lee YM. Crosstalk between angiogenesis and immune regulation in the tumor microenvironment. Arch Pharm Res **2022**;45:401-16

18. Jiang Z, Zhou J, Li L, Liao S, He J, Zhou S*, et al.* Pericytes in the tumor microenvironment. Cancer Lett **2023**;556:216074-

19. Hebbel RP. Blood endothelial cells: utility from ambiguity. J Clin Invest **2017**;127:1613-5

20. Chioh FW, Fong SW, Young BE, Wu KX, Siau A, Krishnan S*, et al.* Convalescent COVID-19 patients are susceptible to endothelial dysfunction due to persistent immune activation. Elife **2021**;10:e64909-

21. Goon PK, Boos CJ, Lip GY. Circulating endothelial cells: markers of vascular dysfunction. Clin Lab **2005**;51:531-8

22. Ng CY, Lee KL, Muthiah MD, Wu KX, Chioh FWJ, Tan K*, et al.* Endothelial-immune crosstalk contributes to vasculopathy in nonalcoholic fatty liver disease. EMBO Rep **2022**;23:e54271-

23. Blann AD, Woywodt A, Bertolini F, Bull TM, Buyon JP, Clancy RM*, et al.* Circulating endothelial cells. Biomarker of vascular disease. Thromb Haemost **2005**;93:228-35

24. Dome B, Timar J, Ladanyi A, Paku S, Renyi-Vamos F, Klepetko W*, et al.* Circulating endothelial cells, bone marrow-derived endothelial progenitor cells and proangiogenic hematopoietic cells in cancer: From biology to therapy. Crit Rev Oncol Hematol **2009**;69:108-24

25. Mehran R, Nilsson M, Khajavi M, Du Z, Cascone T, Wu HK*, et al.* Tumor endothelial markers define novel subsets of cancer-specific circulating endothelial cells associated with antitumor efficacy. Cancer Res **2014**;74:2731-41

26. Shaked Y, Ciarrocchi A, Franco M, Lee CR, Man S, Cheung AM*, et al.* Therapy-induced acute recruitment of circulating endothelial progenitor cells to tumors. Science **2006**;313:1785-7

27. Shaked Y, Henke E, Roodhart JM, Mancuso P, Langenberg MH, Colleoni M*, et al.* Rapid chemotherapy-induced acute endothelial progenitor cell mobilization: implications for antiangiogenic drugs as chemosensitizing agents. Cancer Cell **2008**;14:263-73

28. Kerbel RS, Kamen BA. The anti-angiogenic basis of metronomic chemotherapy. Nat Rev Cancer **2004**;4:423-36

29. Ceradini DJ, Kulkarni AR, Callaghan MJ, Tepper OM, Bastidas N, Kleinman ME*, et al.* Progenitor cell trafficking is regulated by hypoxic gradients through HIF-1 induction of SDF-1. Nat Med **2004**;10:858-64

30. Du R, Lu KV, Petritsch C, Liu P, Ganss R, Passegue E*, et al.* HIF1alpha induces the recruitment of bone marrow-derived vascular modulatory cells to regulate tumor angiogenesis and invasion. Cancer Cell **2008**;13:206-20

31. Ferrari N, Glod J, Lee J, Kobiler D, Fine HA. Bone marrow-derived, endothelial progenitor-like cells as angiogenesis-selective gene-targeting vectors. Gene Ther **2003**;10:647-56

32. Ojeifo JO, Lee HR, Rezza P, Su N, Zwiebel JA. Endothelial cell-based systemic gene therapy of metastatic melanoma. Cancer Gene Ther **2001**;8:636-48
